# Extracellular vesicles secreted by *Brugia malayi* microfilariae modulate the melanization pathway in the mosquito host

**DOI:** 10.1038/s41598-023-35940-9

**Published:** 2023-05-31

**Authors:** Hannah J. Loghry, Hyeogsun Kwon, Ryan C. Smith, Noelle A. Sondjaja, Sarah J. Minkler, Sophie Young, Nicolas J. Wheeler, Mostafa Zamanian, Lyric C. Bartholomay, Michael J. Kimber

**Affiliations:** 1 Department of Biomedical Sciences, College of Veterinary Medicine, Iowa State University, Ames, IA USA; 2 Department of Entomology, College of Agriculture and Life Sciences, Iowa State University, Ames, IA USA; 3 Department of Biology, College of Arts and Sciences, University of Wisconsin-Eau Claire, Eau Claire, WI USA; 4 Department of Pathobiological Sciences, School of Veterinary Medicine, University of Wisconsin-Madison, Madison, WI USA

**Keywords:** Immunology, Immune evasion, Infectious diseases, Infectious diseases

## Abstract

Vector-borne, filarial nematode diseases cause significant disease burdens in humans and domestic animals worldwide. Although there is strong direct evidence of parasite-driven immunomodulation of mammalian host responses, there is less evidence of parasite immunomodulation of the vector host. We have previously reported that all life stages of *Brugia malayi*, a filarial nematode and causative agent of Lymphatic filariasis, secrete extracellular vesicles (EVs). Here we investigate the immunomodulatory effects of microfilariae-derived EVs on the vector host *Aedes aegypti.* RNA-seq analysis of an *Ae. aegypti* cell line treated with *B. malayi* microfilariae EVs showed differential expression of both mRNAs and miRNAs. AAEL002590, an *Ae. aegypti* gene encoding a serine protease, was shown to be downregulated when cells were treated with biologically relevant EV concentrations in vitro. Injection of adult female mosquitoes with biologically relevant concentrations of EVs validated these results in vivo, recapitulating the downregulation of AAEL002590 transcript. This gene was predicted to be involved in the mosquito phenoloxidase (PO) cascade leading to the canonical melanization response and correspondingly, both suppression of this gene using RNAi and parasite EV treatment reduced PO activity in vivo. Our data indicate that parasite-derived EVs interfere with critical immune responses in the vector host, including melanization.

## Introduction

Vector-borne, filarial nematode diseases cause significant health burdens in humans, domestic animals, livestock, and wildlife worldwide. In humans, Lymphatic filariasis (LF) is caused by multiple species of filarial nematodes, including *Brugia malayi,* and is endemic in 72 countries with over 860 million people infected or at risk of infection^1^. Adult parasites reside in the lymphatic vasculature and although often asymptomatic, infection can result in extreme morbidity including lymphangitis, lymphedema (primarily in the extremities), and secondary bacterial infection/dermatitis^2^. Current control strategies rely on mass drug administration programs to disrupt transmission using inadequate anthelmintic drugs that do not effectively kill adult parasites or resolve established infections. The need for new control strategies for filarial nematode diseases is clear, however, progress in developing effective treatments has been stalled by gaps in our understanding of parasite biology and host-parasite interactions. Parasitic filarial nematodes require both an intermediate vector (mosquito) host and a definitive (mammalian) host to complete their life cycle. In either host, the nematode must evade the elicited immune response of the host to develop and establish infection. Various immune evasion strategies have been documented, including manipulation of host immune responses^3^. In mammals, there is direct evidence of this parasite-derived immunomodulation; parasites are capable of expanding regulatory immune cell populations^4–10^, inducing apoptosis in CD4^+^ Th1 cells^11–14^, manipulating pattern recognition receptor (PRR) signaling in those cells that recognize parasite infection^15–21^, and enhancing the general environment of regulatory and anti-inflammatory cytokines, including IL-4 and IL-10^22–24^.

Within the context of the vector host, early studies provided evidence for potential immunomodulation of the melanization immune response by filarial nematodes^25–27^. Melanotic encapsulation, a crucial mosquito innate immune response to the microscopic larval stages of mosquito-infecting parasites, is a core arthropod defense mechanism that prevents nematode growth and reproduction, and eventually leads to their death^28,29^. Melanotic encapsulation involves both the humoral and cellular components of the innate immune response in mosquitoes. Upon recognition of a pathogen, the cellular arm of insect innate immunity drives aggregation of hemocytes, invertebrate immune effector cells, to form a multicellular layer around the invading pathogen. Concurrently, the humoral arm of insect innate immunity initiates melanin production in the hemocytes^30–38^. This process is controlled by the phenoloxidase (PO) cascade, which is initiated when a pathogen associated molecular pattern (PAMP) binds to its pattern recognition receptor (PRR) eliciting a serine protease cascade. This cascade ultimately leads to the activation of a pro-phenoloxidase activating factor, which in turn activates phenoloxidase^39–41^. Phenoloxidase can then oxidize phenols to quinones which are further polymerized to melanin^29^. Death of the parasite is believed to be due to nutrient deprivation, asphyxiation or through the production of toxins such as quinones and other reactive oxygen species produced during melanin production^42,43^. In the mosquito *Aedes aegypti*, this melanization pathway in response to filarial infection is diminished, suggesting parasite manipulation of this critical response^25,27,44^. Supporting the paradigm of parasite-driven modulation of mosquito immune responses, recent studies exploring vector host global transcriptomic changes in response to parasite invasion have identified downregulation of immune-related genes during infection^45–50^.

While there is emerging evidence that vector host immune responses are altered during filarial nematode infection, the potential parasite-derived effectors that might be driving this modulation and their mechanisms of action are undefined. Parasite excretory-secretory products (ESP) are a well-established source of potential effector molecules during mammalian infection. Parasite ESP encompass freely secreted proteins and nucleic acids, as well as extracellular vesicles (EVs), which are membrane-bound structures secreted by both prokaryotic and eukaryotic cells, including filarial nematodes^15,51–54^. They contain complex cargo that can include proteins, small RNA species, and lipids^55,56^ and have been shown to be highly involved in cell-to-cell communication and have roles in various physiological processes^55,57–59^. Although EVs are a newly recognized fraction of parasitic nematode ESP, the cargo of some nematode EVs have been profiled, revealing contents to include protein and small RNA species with predicted immunomodulatory properties^15,51,53,60–68^. There is strong evidence that these EVs are directly involved in immunomodulation of mammalian host responses^4,15,16,51,60,62,63,67,69,70^.

Microfilaria-stage (mf) parasites are consumed during the blood meal and initiate infection of the mosquito. Mf are exposed to the mosquito immune response, including the melanization response^28^, as they migrate from the midgut into the hemocoel and to the thoracic musculature. In potential antagonism, mfs actively secrete EVs^53,60^. We hypothesize that filarial nematode EVs secreted by microfilaria-stage parasites act as effectors to modulate the immune response of the mosquito host. To test this hypothesis, we examined the in vitro modulatory effects of *B. malayi* mf EVs on the global transcriptomic profile of *Aedes aegypti* derived Aag2 cells, an established model for mosquito hemocytes due to their characterized immunocompetence^71,72^. We found that nematode EV treatment drove differential expression of host genes, including a serine protease (AAEL002590) that we identified to have direct involvement in the PO pathway*.* The effect of mf EVs was subsequently investigated in vivo*,* and we show that these mf-derived EVs inhibited PO activity in adult female *Ae. aegypti* at biologically relevant concentrations. These findings provide evidence that parasite-derived EVs are capable of modulating critical vector host immune responses.

## Results

### *B. malayi* mf derived EVs are internalized by Aag2 cells

**Figure 1:**
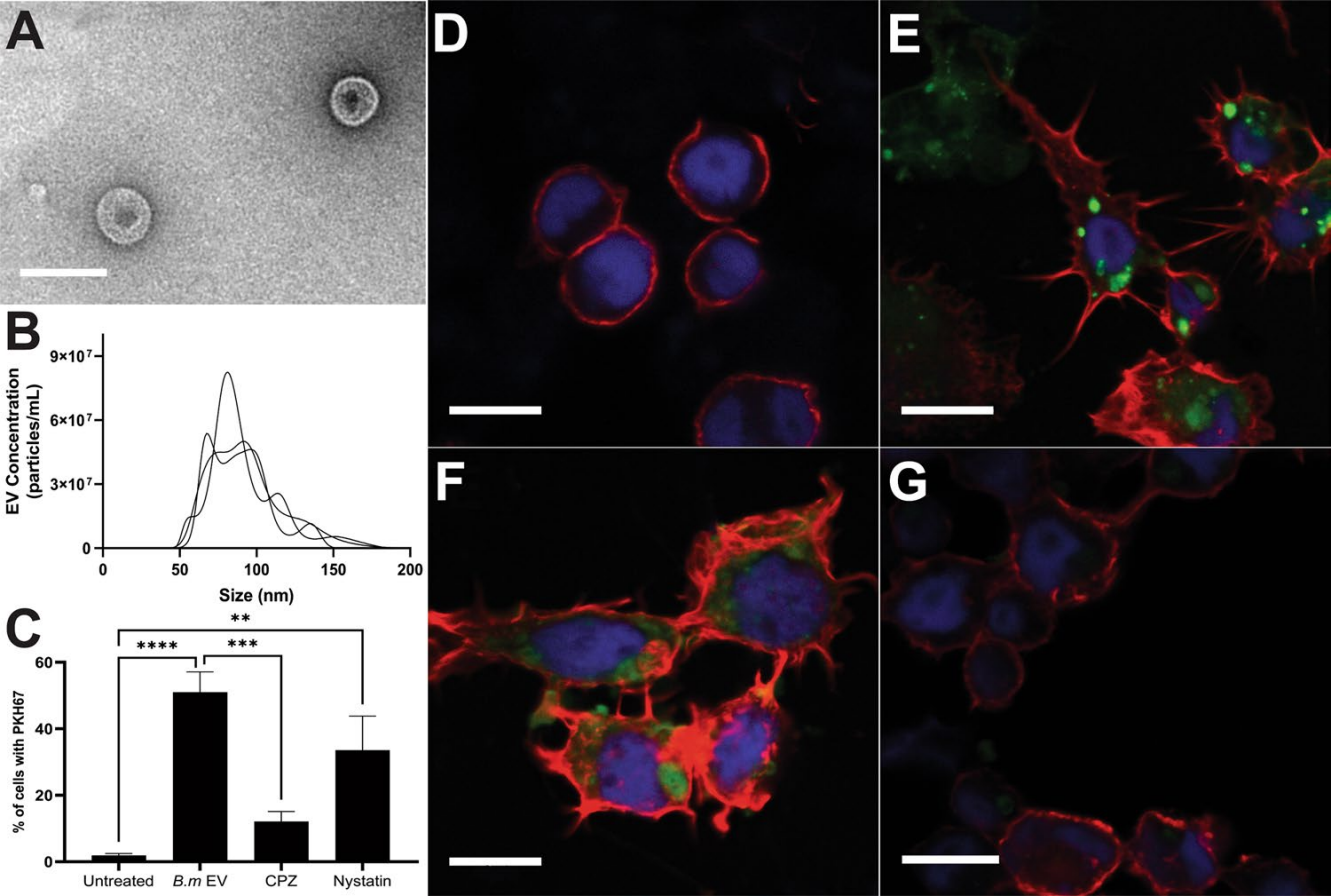
*B. malayi* mf derived EVs are internalized by Aag2 cells. Isolation of *B. malayi* mf EVs was confirmed by TEM (**A**) and size and concentration profile further validated with nanoparticle tracking analysis (**B**). In this representative graph, each line trace indicates a biological replicate EV sample isolated from discrete mf cultures. PKH67-stained *B. malayi* mf EVs were incubated with Aag2 cells for 24 h and EV internalization quantified using a BD Accuri C6 Flow Cytometer (**C**) with 51% of Aag2 cells internalizing *B. malayi* mf EVs. The endocytosis inhibitors chlorpromazine (CPZ) and nystatin were used to determine the mechanism by which these EVs are being internalized. CPZ significantly reduced EV internalization by 39% while nystatin did not. Additionally, EV internalization was assessed by confocal microscopy using a Leica SP5 X MP confocal/multiphoton microscope system. Aag2 cells were labeled with AlexaFluor 647 Phalloidin (red) and DAPI (blue) to visualize nuclei. Aag2 cells internalized PKH67-stained *B. malayi* mf EVs (green) (**E**) as compared to control cells (**D**). Cells treated with CPZ (**G**) showed reduced endocytosis of stained EVs while cells treated with nystatin (**F**) showed continued uptake of EVs. N = 3 experiments (minimum). Mean ± SEM. **P < 0.01, ***P < 0.001, ****P < 0.0001. Scale bar (A) = 150 nm. Scale bar (D-G) = 10 µM.

To confirm both EV secretion by mf and our ability to isolate those EVs from conditioned media, EVs were collected from spent mf-stage culture media and imaged using TEM (Fig. 1A). Particles isolated from spent media exhibited the classic EV-like deflated soccer ball morphology under EM but such structures were absent from unconditioned media. Vesicle size and concentration was described using nanoparticle tracking analysis (NanoSight LM10, Malvern Panalytical, Malvern UK) (Fig. 1B) revealing a discrete vesicle population well within the expected 50–200 nm range (mean size of 92.2 nm). It was also determined that, on average, one million mf secrete 2.6 × 10^9^ EVs in 24 h. To examine the interaction of mf EVs with *A. aegypti* cells, we treated Aag2 cells, an immune-responsive *Ae. aegypti* cell line^71^, with PKH67 stained *B. malayi* mf derived EVs. 24 h after treatment, EV internalization was quantified using flow cytometry (Fig. 1C). Approximately half (51%) of treated Aag2 cells internalized *B. malayi* mf EVs (p < 0.0001, N = 3). For context, we have previously reported 100% of mammalian macrophages treated with labeled *B. malayi* EVs internalize vesicles^53^. To begin to tease apart the mechanism by which mf EVs are being internalized, Aag2 cells were treated with the endocytosis inhibitors chlorpromazine (CPZ) and nystatin prior to EV incubation. CPZ is an inhibitor of clathrin-mediated endocytosis and has been shown to inhibit the function of a key clathrin-mediated endocytic adaptor protein AP2^73,74^. Nystatin binds cholesterol and thus can inhibit caveolin-mediated endocytosis^75^. Pre-treatment with CPZ reduced the number of Aag2 cells that internalized *B. malayi* mf EVs by 39% (p = 0.0003, N = 3). However, pre-treatment with nystatin only reduced internalization by 17% (p = 0.1589, N = 3) and was not statistically significant suggesting EV internalization by Aag2 cells is via clathrin-mediated endocytosis. In addition to quantification by flow cytometry, these cells were also visualized by confocal microscopy. Normal internalization of *B. malayi* mf EVs appeared punctate and focused in discrete areas within the cell rather than being broadly diffused throughout the cytoplasm (Fig. 1D,E). This punctate uptake has been reported before with EVs that are internalized via endocytosis and may reflect the labelled EVs being confined to endosomes within the cytoplasm^76^. In addition, internalization of parasite EVs by murine epithelial cells showed a similar punctate appearance^15^. However, a different phenotype was seen by parasite EVs internalized by murine macrophages and human monocytes where the EVs appeared diffused throughout the cytoplasm^51,53,60^. These differences in internalization may be due to various endocytosis pathways utilized by the heterogenous cell types. Again, our microscopy approach confirmed the flow cytometry data indicating that CPZ, but not nystatin, inhibited the endocytosis of *B. malayi* mf EVs (Fig. 1F,G), supporting the hypothesis that endocytosis of parasitic EVs is clathrin-mediated.

### EV treatment suppresses miRNA expression in LPS-treated Aag2 cells

**Figure 2:**
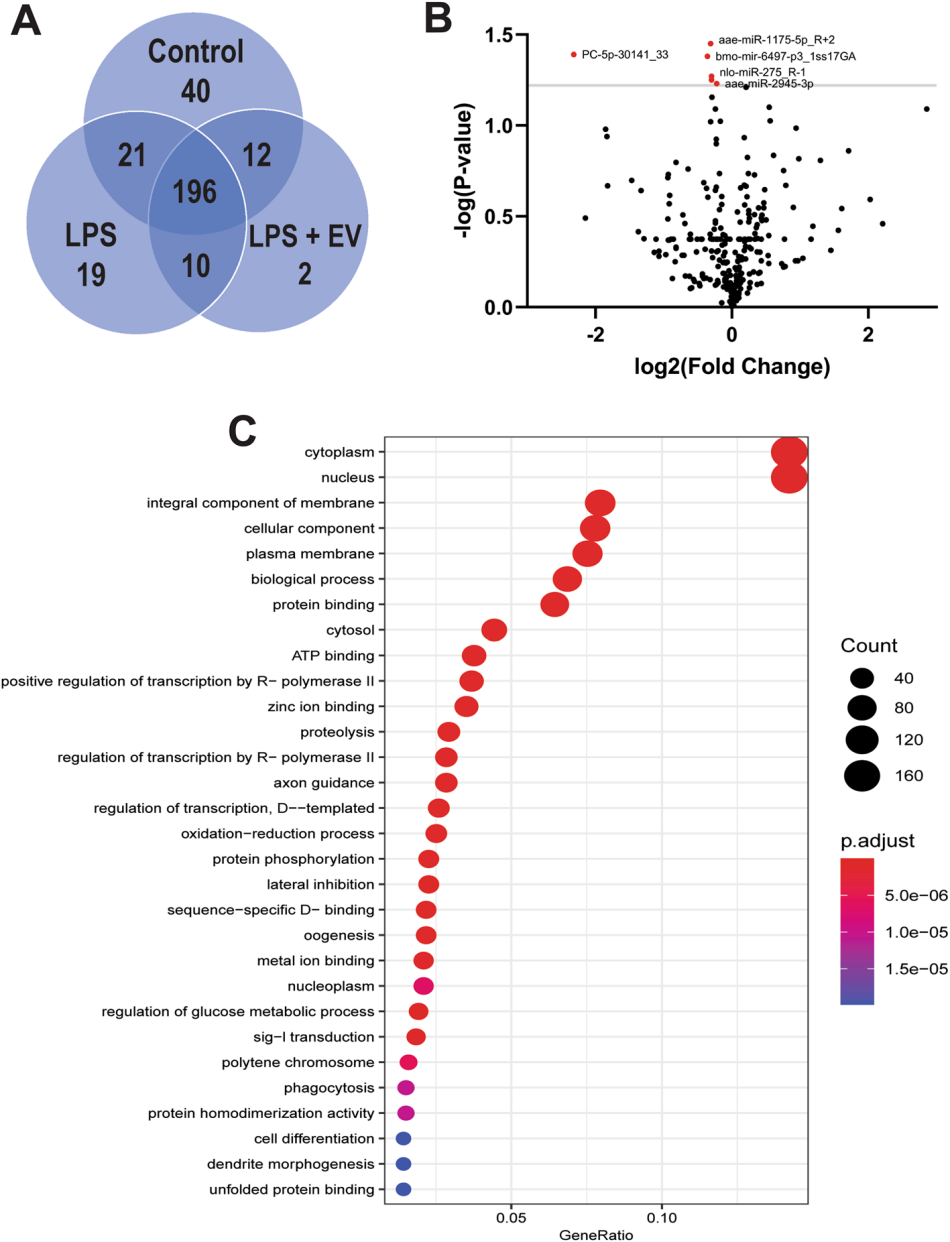
EV treatment suppresses miRNA expression with immune-related targets. miRNA-seq analysis was performed on control, LPS and LPS + EV-treated Aag2 cells. All three treatment groups shared 196 miRNAs while 40, 19 and two miRNAs were unique to control, LPS only and LPS + EV treatment groups respectively (**A**). Volcano plot showing six miRNAs were significantly downregulated between the LPS and LPS + EV treatment groups. No Aag2 miRNAs were upregulated by EV treatment (**B**). Predicted mRNA targets were identified for five out of the six significantly downregulated miRNAs. Gene ontology (GO) analysis of these predicted gene targets identified their role in various physiological processes including proteolysis, signal transduction and regulation of transcription (**C**).

EVs secreted by other *B. malayi* life stages have immunomodulatory activity^51,53^. We therefore hypothesized that EVs secreted by mf parasites would modulate mosquito immune responses. To test this hypothesis we treated Aag2 cells with *B. malayi* mf EVs and examined cellular and molecular phenotypes consistent with immunomodulation. Genetic changes underpin cellular phenotypes so we first described changes in host miRNA expression following EV treatment. Aag2 cells were treated with LPS (500 ng/mL) to stimulate an immune response, followed 12 h later by treatment with either 1X dPBS (control) or *B. malayi* mf EVs to examine the modulatory effects of EV treatment. Our initial screening dosage of 1 × 10^9^ EVs was selected as this is a standard EV dose for cell-based assays throughout the EV literature. Our NTA data would suggest this is a high dose but we felt that by using this concentration, any modulatory effects would be clearly evident. Cells were collected 16 h post-treatment and processed for miRNA sequencing. Of the 300 miRNAs identified, 196 were expressed in all three treatment groups (control, LPS only, and LPS + EV). The control treatment group shared 21 miRNAs with the LPS only treatment group and 12 with LPS + EV while LPS and LPS + EV shared 10 common miRNAs (Supplemental Materials 1). The control, LPS and LPS + EV treatment groups had 40, 19 and two miRNAs that were unique to each treatment group, respectively (Fig. 2A). To assess the ability of *B. malayi* EVs to regulate miRNA expression, we compared miRNA expression between LPS and LPS + EV treatment groups. Six miRNAs were significantly downregulated (log2(fold change) < 0.3 and p-value < 0.05) in LPS + EV as compared to LPS only, including aae-mir-1175, aae-mir-2945, bmo-mir-6497, nlo-mir-275, aae-mir-184, and PC-5p-30141_33 (Fig. 2B). While aae-mir-1175, aae-mir-2945, bmo-mir-6497, nlo-mir-275, aae-mir-184 all had 20% reduction in expression as compared to LPS treated cells, they were among the most statistically significant (p < 0.05, N = 3). PC-5p-30141_33 was the most downregulated miRNA with an 80% reduction in expression as compared to LPS only treated cells (p = 0.0403, N = 3). No mosquito miRNAs were found to be upregulated by parasite EV treatment. Target prediction was conducted on the differentially expressed miRNAs followed by GO analysis of the predicted gene targets. Putative targets were predicted for five out of the six downregulated miRNAs with gene targets of these downregulated miRNAs having roles in proteolysis, regulation of transcription, signal transduction, phagocytosis, and cell differentiation among others (Fig. 2C). Additionally, KEGG analysis revealed that the predicted gene targets are involved in multiple immune-related pathways (Table 1). Gene targets of the downregulated miRNAs are predicted to be involved in common insect immune signaling pathways such as Toll/IMD, MAPK, TGFβ and insulin signaling pathways. Some of these predicted gene targets include AAEL008634, a jnk protein; AAEL010433, a transcriptional co-repressor; AAEL003505, a jun protein; and AAEL013433, a spaetzle-like cytokine. One of the downregulated Aag2 miRNAs, aae-mir-1175, is conserved in *Anopheles gambiae* and is known to be downregulated in *Plasmodium*-infected mosquitoes^77^. In addition, mir-1175 is solely expressed in the mosquito midgut, a critical barrier in parasite development and transmission in the vector host. A similar phenotype was seen in *Ae. aegypti* where aae-mir-1175 was downregulated in mosquitoes infected with dengue virus as compared to uninfected mosquitoes^78^. This provides evidence for a conserved role for this miRNA in immunomodulation across diverse vector-pathogen interactions that enables pathogen migration and development. *Ae. aegypti* infected with *Wolbachia* showed a similar downregulation of aae-mir-2945 when compared to uninfected mosquitoes, providing additional support for downregulation of miRNAs in immunomodulation^79^. The direct roles that downregulating these miRNAs have on insect immune cell responses remain unexplored, but these data suggest that *B. malayi* EVs are capable of modulating the expression of miRNAs, key regulators controlling post-transcriptional gene expression in mosquitoes, including genes involved in mosquito immune signaling pathways.

**Table 1:** KEGG analysis of downregulated miRNA predicted targets.

miRNA	Function of Predicted Target
aae-mir-1175	MAPK Signaling Pathway
	Longevity Regulating Pathway
	Phosatidylinositol Signaling Pathway
	NOD-like Receptor Signaling Pathway
	Wnt Signaling Pathway
	Notch Signaling Pathway
	TGFβ Signaling Pathway
	FoxO Signaling Pathway
	Toll and Imd Signaling Pathway
aae-mir-2945	Phosatidylinositol Signaling Pathway
	MAPK Signaling Pathway
	Wnt Signaling Pathway
	Toll and Imd Signaling Pathway
	Insulin Signaling Pathway
	Neurotrophin Signaling Pathway
	TGFβ Signaling Pathway
	FoxO Signaling Pathway
	Phosatidylinositol Signaling Pathway
	mTOR Signaling Pathway
	Longevity Regulating Pathway
	Ras Signaling Pathway
bmo-mir-6497	Toll and Imd Signaling Pathway
	MAPK Signaling Pathway
	Notch Signaling Pathway
	Longevity Regulating Pathway
	mTOR Signaling Pathway
	Wnt Signaling Pathway
	FoxO Signaling Pathway
	p53 Signaling Pathway
	Hedgehog Signaling Pathway
	Rap1 Signaling Pathway
	Insulin Signaling Pathway
	Neurotrophin Signaling Pathway
PC-5p-30141_33	Hedgehog Signaling Pathway
	MAPK Signaling Pathway
	TGFβ Signaling Pathway
	mTOR Signaling Pathway

KEGG analysis of genes predicted to be targets of six Aag2 miRNAs significantly downregulated by parasite EV treatment. This analysis revealed an enrichment of immune signaling pathways, including the immune signaling pathways Toll/IMD, MAPK, TGFβ and insulin signaling.

### EV treatment modulates gene expression in LPS-treated Aag2 cells

**Figure 3:**
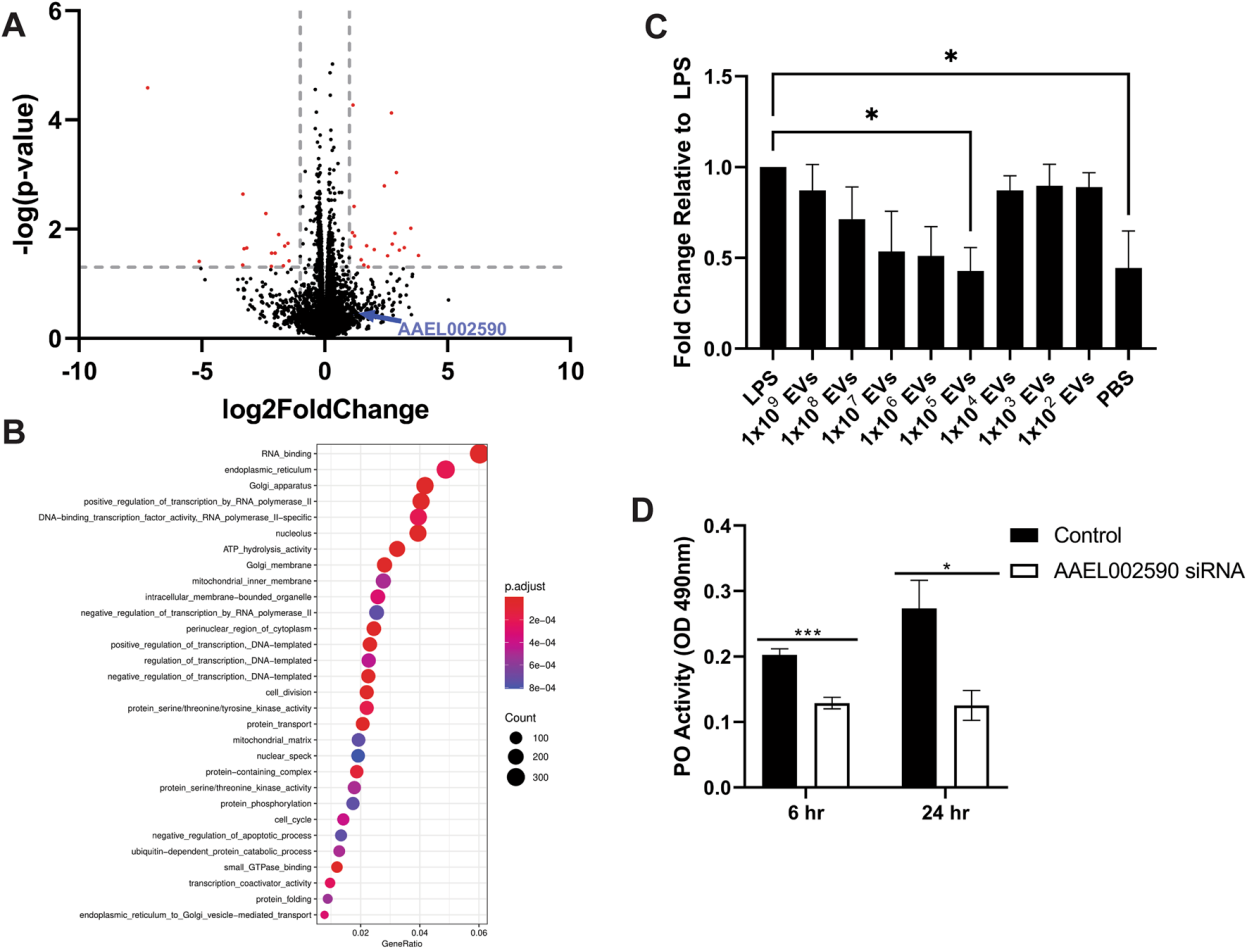
EVs released by *B. malayi* microfilariae modulate vector host gene expression. A volcano plot showing differentially expressed genes between LPS and LPS + high dose EV treatment groups (**A**) including a putative serine protease (AAEL002590). GO analysis was conducted on differentially expressed genes. No significant enrichment in GO terms was identified for upregulated genes but significant enrichment was identified for the downregulated genes. Enriched GO terms were associated with regulation of transcription and protein phosphorylation (**B**). mRNA-seq was validated using qPCR. As EV concentrations were tittered down to more biologically relevant concentrations (1 × 10^5^ *B. malayi* mf EVs), it was revealed that AAEL002590 was in fact significantly downregulated at these concentrations, conflicting with the initial mRNA-seq screen (**C**). RNAi suppression of AAEL002590 in Aag2 cells inhibited phenoloxidase activity at both 6 and 24 h post-treatment (**D**) indicating AAEL002590 is involved in the PO pathway and supporting our hypothesis that parasite EVs modulate mosquito immune responses. N = 3 (minimum). Mean $\pm$ SEM. * P < 0.05, ***P < 0.001.

After observing downregulation of miRNA expression, we hypothesized EV treatment would also impact mRNA expression in Aag2 cells. To test this hypothesis, mRNA-seq analysis was conducted on Aag2 cells with treatment groups and EV dosing as described in the miRNA experiments. This analysis revealed modest differential mRNA expression between the LPS and LPS + EV treatment groups (Fig. 3A). Our initial conditions that needed to be met for a gene to be further investigated included more than a twofold change in expression, a p-value < 0.05 and for that gene product to have an annotated function consistent with response to pathogen. Unfortunately, while many genes were identified to be differentially regulated (log2(fold change) > 1or log2(fold change) < −1), this up- and down-regulation was only statistically significant (p < 0.05) for a small cohort of genes. Interestingly, while evaluating genes within our fold change threshold, but outside of the statistically significant threshold, we identified that this expanded pool of differentially regulated genes was enriched in transcripts encoding proteins that are strong candidates for immunomodulation, including a cohort of proteases with potential involvement in the melanization pathway (Table 2). The full list of differentially expressed genes can be found in Supplemental Materials 2.

**Table 2:** Immune related differentially expressed genes.

Upregulated			
Gene	Annotation from Vectorbase	BLAST Derived Homology	Function
AAEL020291		MAP3K15	MAPK/ERK Signaling
AAEL002731	Serpin 14		Melanization
AAEL002301	Serine Protease		Melanization
AAEL020706		Serine Protease-like	Melanization
AAEL009722	Clip-Domain Serine Protease		Melanization
AAEL002624	Serine Protease		Melanization
AAEL003253	Clip-Domain Serine Protease		Melanization
AAEL020362		PPAF-3	Melanization
AAEL003713	Leucine-Rich Transmembrane Protein	Toll-like Receptor 4	Toll Signaling Pathway
AAEL002590	Serine Protease	PPAF-2	Melanization
AAEL001744	Jnk interacting protein		Jnk Signaling Pathway

Downregulated			
Gene	Annotation/BLAST Result		Function
AAEL013496	Prophenoloxidase 8		Melanization
AAEL000038	CLIP-Domain Serine Protease		Melanization
AAEL003389	Attacin		Antimicrobial Peptide
AAEL019938	Superoxide Dismutase		Antioxidant

GO enrichment analysis was conducted on all up- or down-regulated mRNAs. No significant GO terms were enriched for the upregulated genes perhaps due to their uncharacterized nature or lack of annotation in the *Ae. aegypti* genome. Genes downregulated following EV treatment were enriched for GO terms associated with regulation of transcription and protein phosphorylation (Fig. 3B). These findings add support to the hypothesis that EVs have immunomodulatory functions. In addition to immunomodulation indicated by differential expression of genes involved in various immune functions the GO analysis might indicate that *B. malayi* mf-derived EVs can drive modulation through regulation of transcription and alterations in protein phosphorylation.

Whilst several of the differentially regulated genes have established roles in mosquito immune response pathways, others do not. One such differentially regulated gene, AAEL002590, is annotated as a putative serine protease in VectorBase, a VEuPathDB resource^80^. BLAST analysis revealed that AAEL002590 has 65% identity to a known pro-phenoloxidase activating factor (PPAF) in *Culex quinquefasciatus* (CPIJ017796) as annotated in VectorBase*.* This homology might suggest a potential role for AAEL002590 in the canonical melanization immune response. We therefore chose to center our qPCR validation of the mRNA-seq analysis on AAEL002590 because (1) it was identified as a differentially regulated gene, and (2) it may have previously unrecognized functions in the mosquito melanization pathway. To validate the mRNA-seq data, Aag2 cells were stimulated with LPS to elicit an immune response and then followed with treatment of serial dilutions of *B. malayi* mf EVs. AAEL002590 expression was assayed using qPCR. Using this approach, treatment of Aag2 cells with 1 × 10^9^ EVs, the same dose used for RNA-seq, did not significantly dysregulate AAEL002590 expression compared to LPS treated cells (p = 0.9757, N = 3). However, 1 × 10^9^ EVs may not be a biologically relevant dose. We define biologically relevant concentrations as the range of EVs that would be expected to be present in a mosquito after a blood meal. While the number of mf taken up by a mosquito during a blood meal varies on the microfilariae density in the blood of the host, the approximate mean number of mf taken up by a mosquito has been measured between 1 and 300 mf with most taking up approximately 40 mf^81–83^. In addition, it has been shown that individual *B. malayi* mf secrete, on average, 4000 EVs in 24 h^54^. These data provide the approximate range of EVs that would be present in a mosquito within 24 h of a blood meal to be between 1 × 10^5^ and 1 × 10^6^. As we tittered down EV concentrations to biologically relevant concentrations, it was found that expression of AAEL002590 was significantly downregulated by 57% after treatment with 1 × 10^5^ *B. malayi* mf EVs (p = 0.0223, N = 3) (Fig. 3C). EV treatment completely abrogated the LPS stimulation of AAEL002590 to basal levels observed in non-LPS treated Aag2 cells (p = 0.0271, N = 3). The discrepancy between the high EV dose used in our RNAseq experiment and the low dose that was validated to downregulate AAEL002590 in vitro suggests that the miRNA and mRNA seq data are likely a huge underestimation of the transcriptional effects of EV treatment. Investigating transcriptional changes due to EVs at the lower dose is an obvious next step to further evaluate the effects of *B. malayi* mf derived EVs.

Since AAEL002590 was identified as a putative serine protease with 65% identity to a *C. quinquefasciatus* PPAF, we wanted to investigate whether this gene is involved in PO activation. RNAi was used to knockdown AAEL002590 expression in Aag2 cells in vitro. A time course experiment showed 79% suppression of AAEL002590 transcript 24 h post-RNAi treatment (p = 0.0012, N = 3) (Supplemental Materials 3). To investigate whether AAEL002590 was involved in PO activation, Aag2 cells were treated with duplexed siRNA or scrambled siRNA as a control for 24 h and then analyzed for PO activity. Basal PO activity was inhibited by 36% following AAEL002590 suppression at 6 h (p = 0.0002, N = 3) and a 54% inhibition at 24 h compared to controls (p = 0.018, N = 3) (Fig. 3D). While LPS treatment has been used to induce an immune response in Aag2 cells previously^71^ and was successful in inducing an immune response at the transcriptional level in Aag2 cells as evident by our gene expression experiments (Fig. 3C), LPS did not strongly induce the PO cascade in vitro at a protein level (Supplemental Materials 4).

### In vivo phenoloxidase activity is inhibited by EV treatment

**Figure 4:**
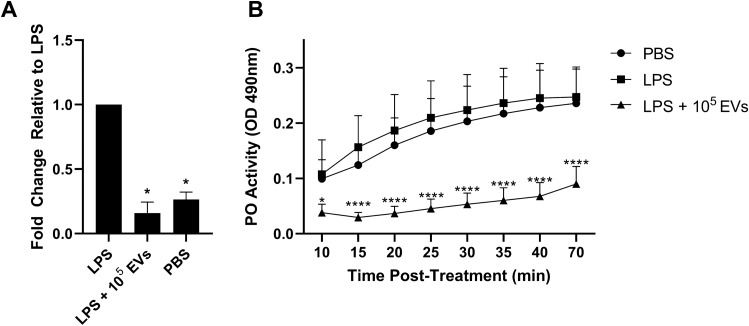
Phenoloxidase activity is inhibited by EV treatment. Validation of AAEL002590 downregulation in vivo was investigated by injection of adult female *Ae. aegypti* mosquitoes (LVP) with serial dilutions of *B. malayi* mf EVs after initial treatment with LPS. 1 × 10^5^ EVs significantly downregulated AAEL002590 as compared to LPS-only (**A**). Phenoloxidase (PO) activity was measured using hemolymph perfused from treated mosquitoes. Treatment of adult female *Ae. aegypti* with 1 × 10^5^ mf EVs inhibited PO activity as compared to LPS-only at all time points (**B**). N = 3 (minimum). Mean $\pm$ SEM. *P < 0.05, ****P < 0.0001.

Having validated that parasite EV treatment downregulates AAEL002590 and PO activity in vitro, we wanted to determine if this phenotype was recapitulated in vivo. Adult female *Ae. aegypti* Liverpool strain (LVP) mosquitoes were injected with LPS (1 mg/mL) followed by injection with 1 × 10^5^ mf EVs or 1X dPBS 6 h later. Mosquitoes were incubated for 24 h and then AAEL002590 expression was assayed by qPCR. LPS treatment increased expression of AAEL002590 by 174% (p = 0.012, N = 3) in mosquitoes, but EV treatment completely abrogated this increase in AAEL002590 expression, returning it to basal levels (p = 0.02, N = 3) (Fig. 4A). To assess whether this downregulation of LPS-induced AAEL002590 expression impacted the PO pathway, hemolymph was collected and PO activity was analyzed as previously described^84^. PO activity was significantly inhibited (ranging from 64 to 80%) in hemolymph from mosquitoes injected with 1 × 10^5^ mf EVs at all experimental time points (p < 0.05 or p < 0.0001, N = 3) (Fig. 4B). While LPS treatment did not increase PO protein levels as highly as expected in these ex vivo experiments, the pronounced inhibition on PO activity after EV treatment is compelling, suggesting that *Brugia* mf EVs suppress PO activation in vitro and in vivo.

## Discussion

While parasite-driven immunomodulation of the mammalian host is well accepted, evidence of immunomodulation of the vector host is lacking. In the current study we have shown that extracellular vesicles (EVs) secreted into the extracellular environment by mf stage *B. malayi* parasites elicit transcriptional changes in an immune-responsive mosquito cell line and in vivo. This study identified multiple genes with potential roles in the phenoloxidase (PO) cascade and other immune responses that were differentially expressed upon EV treatment. While not all these genes were investigated in more depth here, the recognition that AAEL02590 is downregulated by parasite EVs leading to inhibition of the PO pathway in vivo establishes a mechanism for parasite-driven vector immunomodulation and underscores the need for further exploration of this process. In addition, the identification of genes targeted by parasites during infection over millions of years of co-evolution is likely to provide critical new insight into essential elements of mosquito immune responses.

**Figure 5:**
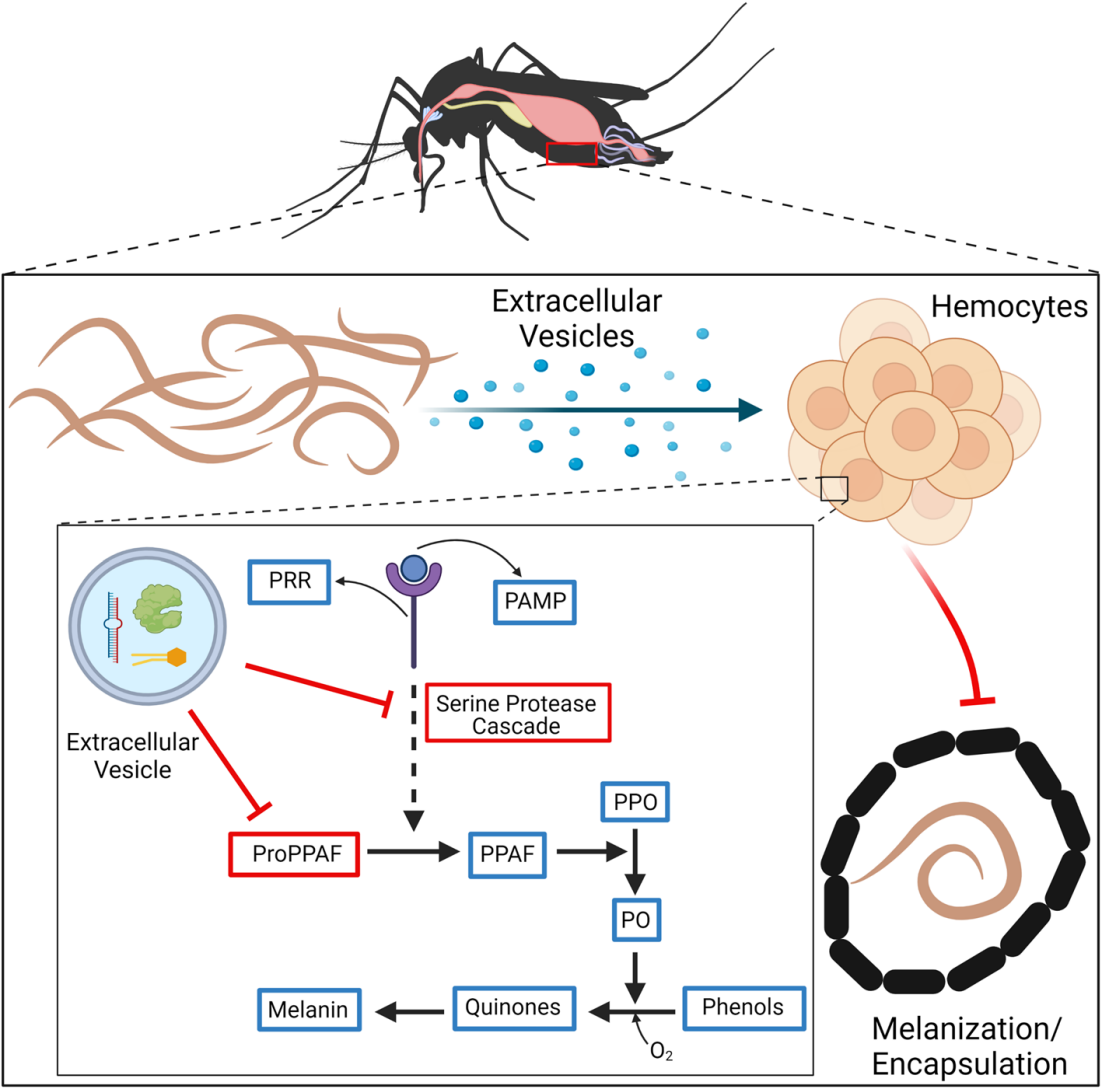
A model for how *B. malayi* microfilariae EVs interfere with the PO cascade and melanization. Melanotic encapsulation is a common insect defense mechanism against parasites. Upon recognition of a parasite, hemocytes aggregate forming a multicellular layer that deposits a melanin-enriched capsule around the invading parasite. Melanin production is controlled by the phenoloxidase (PO) cascade, which through a series of interdependent reactions, leads to the activation of PO that oxidizes phenols to quinones, which are further polymerized to melanin. Death of the parasite is believed to be due to nutrient deprivation, asphyxiation, or through the production of toxins such as quinones and other reactive oxygen species produced during melanin production. *B. malayi* microfilariae-derived extracellular vesicles downregulate a serine protease that functions either at the serine protease cascade or as a PPAF, either way interfering with the production of PO and thus inhibiting melanization of invading parasites. Image was created with Biorender.com.

Melanotic encapsulation, promoted by the PO cascade, is a fundamental mosquito defense mechanism against filarial parasites^28,29^. We have found that parasite EV exposure downregulates a putative *Ae. aegypti* serine protease orthologous to a *Cu. quinquefasciatus* prophenoloxidase activating factor (PPAF) (CPIJ017796)*.* Further experimentation is needed to determine if this serine protease is indeed a true PPAF or if it is a serine protease involved in an upstream cascade that activates pro-PPAF but in either case, our data provide evidence that parasite-derived EVs are effector structures in immunomodulation of vector hosts with the ability to interfere with critical host immune responses (summarized Fig. 5). In these experiments we focused on Aag2 cells as a model for mosquito hemocytes, but we recognize that EVs can be effecting other tissues/cell types that are involved in the melanization response or involved in cellular communication between immune tissues such as the fat body^85,86^. While our data provide novel mechanistic evidence for modulation of the host melanization immune response, previous work has hinted at the importance of regulating the melanization pathway for pathogens. Christensen and LaFond (1986) were the first to provide evidence for parasite-derived modulation of the melanization response, showing that *B. pahangi* infected *Ae. aegypti* had reduced ability to melanize when challenged with intrathoracic inoculation of new *B. pahangi* mf^25^. Beerntsten et al. showed that *B. pahangi* did not have to come in contact with host antigen in order to evade melanization, suggesting that the parasites were producing effectors capable of modulating this response in some way^27^. This led to a transcriptomic study that identified that *B. pahangi* secrete factors such as serpins that were postulated to be involved in modulation of melanization^26^. In addition, targeting of the melanization and encapsulation immune response is a common phenotype seen in infections of *Galleria mellonella* with the parasitic nematode *Steinernema carpocapsae.* Studies have shown that a trypsin-like serine protease secreted by *S. carpocapsae* can inhibit PO activity in vitro and affects the morphology of *S. capocapsae* hemocytes thus inhibiting spreading behaviors, a feature necessary for encapsulation^87^. Further, a secreted chymotrypsin protease from *S. carpocapsae* has also been shown to inhibit PO activity and encapsulation of *G. mellonella* hemocytes both in vitro and in vivo^88^. *Brugia* are known to actively secrete a number of proteases, some of which may be involved in modulating the melanization response; indeed, a cathepsin L-like protease is abundantly found in the EVs of infective third-stage larvae isolated from *Ae. aegypti*^51^ that is essential to parasite survival within the mosquito^89^. An important next step will be to characterize the cargo of *B. malayi* mf EVs to identify those effector molecules responsible for PO pathway downregulation. As this work continues, it will be essential to consider that the modulatory molecules may not be proteins. We have shown that filarial nematode EVs also contain a diverse miRNA cargo^51^ and secreted EVs represent a way that effector miRNAs can be released from the parasite and protected during trafficking to host cells, where they might downregulate immune pathways at the genetic level.

To this end, several transcriptomic studies have looked at the global transcriptional changes that occur in the mosquito host during parasite infection^45–50^. Many of these studies also identified parasite-derived downregulation of mosquito host serine proteases at the transcriptional level. A study conducted on *B. malayi*-infected *Armigeres subalbatus* showed that there was a significant reduction in expression of multiple serine protease genes during the first 24 h of infection^45^. This correlates with the time frame that EV-secreting mfs would be migrating from the midgut, through the hemocoel and to the thoracic musculature, and the timeframe seen in our studies. While *B. malayi* do not effectively develop to infective L3 stage parasites in *A. subalbatus*, it still provides evidence for host transcriptional changes during early stages of infection. Similar trends were observed in *B. malayi* infected *Ae. aegypti* where there was evidence for parasite derived alteration in expression of genes involved in blood digestion and immune function including specific downregulation of serine protease genes^47^, providing broad evidence for parasite immunomodulation in a compatible vector model. In addition, a study looking at transcriptional changes in both *B. malayi* and *Ae. aegypti* during the course of infection saw that between 2–4 days post-infection, an *Ae. aegypti* serine protease gene, AAEL002590, was downregulated in infected mosquitoes^50^. This downregulation occurring 2–4 days post-infection correlates with our study as 2 days post-infection broadly aligns with the mf to L1 molt within the thoracic muscles, although some mf will still be present^47^. It is also important to note that while our data shows downregulation occurring as early as 24 h post-treatment, this may be due to the fact that we are injecting isolated EVs and not infecting with live parasites. Many of these transcriptomic studies also identified downregulation of CLIP serine proteases or prophenoloxidase enzymes^47–49^, key components involved in the PO cascade and melanization immune response, providing additional corroboration for our observation that *B. malayi* mf EVs are interfering with this immune response. While our study provides a mechanism for parasite-derived transcriptional changes of the host, all these transcriptional studies provide a substrate for further studies aimed at better understanding mosquito immune responses. Paying particular attention to those mosquito genes that filarial nematode parasites have selected to suppress over millions of years of the host-parasite interaction may reveal the most critical pathways and proteins to exploit for insecticides or novel transmission control strategies.

Our understanding of the filarial nematode-vector interface is incomplete, but the data described in this paper helps to begin addressing this knowledge gap and may even seed the identification of novel targets that could contribute to better controlling filarial nematode diseases. Identifying targets at the vector stage of parasite development may stop transmission of the causative agents of filarial diseases and may provide insight into control strategies for other non-filarial, vector-borne diseases. Any mechanism that disrupts the vector-parasite interaction and skews the balance in favor of the vector is likely to prevent infection, parasite development, and ultimately disease transmission.

## Materials and methods

### Cell culture

The immunocompetent *Aedes aegypti*-derived Aag2 cell line was cultured in Schneider’s *Drosophila* medium supplemented with 10% heat-inactivated, fetal bovine serum and 1% Penicillin/Streptomycin (all Thermo Fisher Scientific, Waltham, MA, USA) in normoxic conditions at 28 °C. Aag2 cells were a kind gift to LCB from Dr. Ann Fallon, University of Minnesota.

### Parasite culture and maintenance

*Brugia malayi* parasites were obtained from the NIH/NIAID Filariasis Research Reagent Resource Center (FR3) at the University of Georgia, USA. Microfilaria were washed according to FR3 protocols upon arrival at Iowa State University. Briefly, microfilariae were centrifuged at 720 × g for 10 min at room temperature to pellet parasites. Transport media [RPMI with Penicillin (2000 U/mL) and Streptomycin (2000 µg/mL)] was aspirated and the parasite pellet resuspended in 1X dPBS (Thermo Fisher Scientific). The parasite suspension was overlaid onto 10 mL of Histopaque-1077 (Sigma Aldrich, St. Louis, MO, USA) and centrifuged at 2000 rpm for an additional 15 min. The supernatant was aspirated and parasite pellet washed with 1X dPBS twice for 5 min each wash. After washing, the supernatant was aspirated and 3 mL of cell culture grade water (Cytiva, Marlborough, MA, USA) was added to the remaining pellet to lyse red blood cells (RBCs). Immediately following RBCs lysis, 10 mL 1X dPBS was added and parasites centrifuged for an additional 5 min then washed one final time in 1X dPBS. Microfilariae were then resuspended in worm culture media (RPMI with 10 mM HEPES, 2 mM L-glutamine, Penicillin (2000 U/mL), Streptomycin (2000 µg/mL), and 1% w/v glucose [all Thermo Fisher Scientific]) and cultured at 37 °C with 5% CO_2_ for 5–7 days. Parasite motility and morphology were used as an indicator of parasite viability. Parasite viability and morphology were manually evaluated under a microscope daily. Briefly, viable parasites are motile, appear dense and curvy in shape while non-viable parasites are translucent, have a straight morphology and are non-motile. Spent media was collected every 24 h and retained for EV isolation as long parasites appeared viable.

### Mosquito rearing

*Ae. aegypti* (Liverpool strain) mosquitoes were reared at 27 °C and 80% relative humidity with a 14:10 h light/dark period. Larvae were fed a 50:50 diet of Tetramin ground fish flakes (Tetra, Melle, Germany) and milk bone dog biscuits. Adults were maintained on a 10% sucrose solution as previously described^90^. All experimental techniques were performed on cohorts of 4–6 days old adult female mosquitoes.

### EV isolation, quantification and imaging

EVs were isolated from spent culture media via differential ultracentrifugation as previously described^51,53,54^. Briefly, media was filtered through 0.2 μm PVDF filtered syringes (GE Healthcare, Chicago, IL, USA) and centrifuged at 120,000 × *g* for 90 min at 4 °C. The supernatant was decanted leaving approximately 1.5 mL media to ensure that the EV pellet was not disrupted. The retained media and pellet were filtered through a PVDF 0.2 μm syringe filter and centrifuged at 186,000 × *g* for a further 2 h at 4 °C. The size profile and concentration of EVs in the isolated sample were quantified using nanoparticle tracking analysis (NTA; NanoSight LM10, Malvern Instruments, Malvern, UK). EV integrity and morphology were confirmed using transmission electron microscopy (TEM). Briefly, a 2 µL aliquot of EV preparation was placed onto a carbon film grid (Electron Microscopy Sciences, Hatfield, PA, USA) for 1 min. The drop was wicked to a thin film and 2 µL of uranyl acetate (2% w/v final concentration) was immediately applied for 30 s, wicked, and allowed to dry. Images were taken using a 200 kV JEOL 2100 scanning and transmission electron microscope (Japan Electron Optics Laboratories, LLC, Peabody, MA) with a Gatan OneView camera (Gatan, Inc. Pleasanton, CA).

### EV internalization by Aag2 cells

Methods were based on protocols previously described^53^, but modified for optimal imaging of the Aag2 cell line. 3 × 10^5^ Aag2 cells were seeded on an 18 mm, #1 thickness, poly-D-lysine coverslip (Neuvitro, Vancouver, WA) in a 12-well plate (Thermo Fisher Scientific) and cultured at 28 °C overnight. Between 5 × 10^8^ and 1 × 10^9^ isolated EVs were stained with PKH67 (Sigma Aldrich, St. Louis, MO) according to the manufacturer’s instructions. Confluent Aag2 cells were treated with 3.5 × 10^7^ stained EVs and incubated for 24 h at 28 °C. EV uptake was visualized with immunocytochemistry. Media was removed and cells were washed with 1X dPBS and fixed in 4% paraformaldehyde (Electron Microscopy Sciences) for 15 min at room temperature. Following three 1X dPBS washes at room temperature, cells were incubated with 1:300 Alexa Fluor 647 phalloidin (Thermo Fisher Scientific) for 45 min at room temperature followed by three washes of 1X dPBS for 5 min each. Cells were incubated with 300 nM DAPI (Thermo Fisher Scientific) for 5 min at room temperature followed by two washes in 1X dPBS. Coverslips were mounted using Flouromount aqueous mounting media (Sigma Aldrich) and visualized by a Leica SP5 X MP confocal/multiphoton microscope system (Leica Microsystems Inc., Buffalo Grove, IL, USA).

Concurrently, EV internalization was quantified using flow cytometry. 3 × 10^5^ cells were seeded per well of a 12-well plate and incubated at 28 °C overnight. Cells were incubated with 3.5 × 10^7^ PKH67 stained EVs for 24 h at 28 °C. Cells were washed in 1X dPBS and collected into polystyrene FACS tubes (Thermo Fisher Scientific). Cells were fixed in 4% paraformaldehyde for 20 min and washed with FACS buffer (1X dPBS supplemented with 1% BSA and 0.1% NaN_3_). Cells were resuspended in 400 µL FACS buffer and analyzed with a BD Accuri C6 Flow Cytometer (BD Biosciences, San Jose, CA). For endocytosis inhibition assays, Aag2 cells were treated with a final concentration of either 30 µM chlorpromazine or 15 µM nystatin (Thermo Fisher Scientific). Following a two-hour incubation, media was changed and cells treated with 3.5 × 10^7^ *B. malayi* mf EVs, incubated for 24 h and then collected for confocal microscopy and flow cytometry as described above. A minimum of three biologically distinct experiments, including biological distinct EV preparations, were performed for both confocal microscopy and quantification by flow cytometry.

### miRNA-seq analysis

Three biologically distinct microRNA (miRNA) samples, defined by both biological distinct cell passages and EV batches, were prepared for miRNA sequencing and performed by LC Sciences. The total RNA quality and quantity were analyzed by Bioanalyzer 2100 (Agilent Technologies, Santa Clara, CA) with RIN number > 7.0. Small RNA libraries were prepared using 1 µg of total RNA and the TruSeq Small RNA Sample Prep Kits (Illumina) according to manufacturer’s instructions. Single-end sequencing was performed on an Illumina Hiseq 2500 (Illumina) according to manufacturer’s instructions. Raw reads were subjected to an in-house program, ACGT101-miR (LC Sciences), to remove adapter dimers and junk, low complexity and common non-target RNA families (rRNA, tRNA, snRNA, snoRNA) and repeats. Remaining unique sequences with length 18–26 nucleotides were mapped to specific species precursors in miRBase 22.0^91–96^ and by BLAST search^97^ to identify known miRNAs and novel 3p- and 5p- derived miRNAs with their genomic location. Length variation at both 3′ and 5′ ends and one mismatch inside of the sequence were allowed in the alignment. The unique sequences mapping to specific species mature miRNAs in hairpin arms were identified as known miRNAs. The unique sequences mapping to the other arm of known specific species precursor hairpin opposite to the annotated mature miRNA-containing arm were considered to be novel 5p- or 3p derived miRNA candidates. Hairpin RNA structures of unmapped sequences were predicted from the flanking 80 nucleotide sequences using RNAfold^98^. The criteria for secondary structure prediction included number of nucleotides in one bulge in stem (≤ 12), number of base pairs in the stem region of the predicted hairpin (≥ 16), cutoff of free energy (kCal/mol ≤ -15), length of hairpin (up and down stems + terminal loop ≥ 50), length of hairpin loop (≤ 20), number of nucleotides in one bulge in mature region (≤ 8), number of biased errors in one bulge in mature region (≤ 4), number of biased bulges in mature region (≤ 2), number of errors in mature region (≤ 7), number of base pairs in the mature region of the predicted hairpin (≥ 12) and percent of mature sequences in stem (≥ 80). To predict the genes targeted by most abundant miRNAs, two computational target prediction algorithms TargetScan^99–101^ and Miranda 3.3a^102^ were used to identify putative miRNA binding sites. Finally, the data predicted by both algorithms were combined and the overlaps calculated. The R package, enrichplot, was used to visualize GO term enrichment from the 2,143 predicted target transcripts of the statistically downregulated miRNAs. These transcripts were trimmed to include only transcripts of the primary isoform of the gene and with p-values < 0.05. The KEGG pathway database was used to analyze the function of the predicted gene targets of the differentially expressed miRNAs^103–106^.

### mRNA-seq analysis

1 × 10^5^ Aag2 cells were seeded in each well of a 96-well plate (Corning Inc, Corning, NY, USA) and incubated overnight at 28 °C. The following day, culture media was changed and cells were treated with either lipopolysaccharide (LPS) (500 ng/mL) to stimulate an immune response in vitro or 1X dPBS as a negative control. Cells were incubated for an additional 12 h at 28 °C after which, culture media was changed and cells treated with 1.1 × 10^9^ parasite EVs per well. Cells were then incubated for a further 16 h at 28 °C before collection and storage in Trizol (Thermo Fisher Scientific) ahead of RNA extraction. Briefly, cells in Trizol were mixed with chloroform (0.2 mL chloroform per mL Trizol) and shaken vigorously for 20 s. Samples were allowed to sit at room temperature for 3 min and then centrifuged at 10,000 × *g* for 18 min at 4 °C. The aqueous phase was collected, and an equal volume of 100% ethanol was added. RNA was then purified and collected using a RNeasy Mini Kit (Qiagen, Hilden, Germany) according to manufacturer’s instructions.

Three biologically distinct RNA samples, defined by both biological distinct cell passages and EV batches, were prepared for mRNA-seq and performed by LC Sciences (Houston, TX). Total RNA quantity and purity were analyzed using an RNA 6000 Nano LabChip Kit and a Bioanalyzer 2100 (Agilent, Santa Clara, CA). High quality RNA samples with RIN number > 7 were used to construct the sequencing library. mRNA was purified from total RNA (5 µg) using Dynabeads Oligo (dT)(Thermo Fisher Scientific) with two rounds of purification. Following purification, mRNA was fragmented into short fragments using a NEB Next Magnesium RNA Fragmentation Module (New England Biolabs, Ipswich, MA, USA) at 94 °C for 5–7 min. Cleaved RNA fragments were reverse transcribed to cDNA by Superscript II Reverse Transcriptase (Thermo Fisher Scientific) and the resulting cDNA used to generate U-labeled second-stranded DNA using *E. coli* DNA polymerase I, RNase H (both New England Biolabs) and dUTP Solution (Thermo Fisher Scientific). An A-base was added to the blunt ends of each strand, preparing them for ligation to the indexed adapters. Each adapter contained a T-base overhang for ligating the adapter to the A-tailed fragmented DNA. Dual-index adapters were ligated to the fragments, and size selection was performed with AMPureXP beads (Beckman Coulter, Brea, CA, USA). U-labeled second-stranded DNAs were treated with heat-labile UDG enzyme (New England Biolabs), and ligated products were amplified with PCR by the following conditions: initial denaturation at 95 °C for 3 min; 8 cycles of denaturation at 98 °C for 15 s, annealing at 60 °C for 15 s, and extension at 72 °C for 30 s; and final extension at 72 °C for 5 min. The average insert size for the paired-end libraries was 300 bp (± 50 bp). Paired-end sequencing was performed on an Illumina Hiseq 4000 (Illumina, San Diego, CA, USA). Reads were adapter and quality trimmed using Trimmomatic^107^. HISAT2^108^ and StringTie^109^ were used to align surviving reads to the *B. malayi* reference genome (WormBase ParaSite version 12.4)^110,111^ and to the *Ae. aegypti* reference genome (VectorBase release 47)^112^ to produce raw counts for annotated genes.

RNAseq reads were evaluated for quality with Fastqc 0.11.7^113^ and Multiqc 1.5^114^. Reads were subjected to trimming using Trim galore 0.6.4^115^, and then aligned to the genome using Hisat2 2.2.0^116,117^. The genome for Aedes aegypti from VectorBase-58^118^ was downloaded from Vectorbase^119^, while the *Brugia malayi* genome (PRJNA10729.WBPS17)^120^ was downloaded from Wormbase Parasite^121^. Samtools 1.10.2^122^ was used for sorting and bam conversion, while FeatureCounts from Subread 1.6.0^123^ was used to allocate reads to genes. Deseq2 1.24.0^124^ was used to perform differential expression and to generate PCA plots. Functional information on protein annotations was downloaded from UniProt on August 31, 2022^125^. ClusterProfiler 4.6.0^126^ was used to generate ontological enrichments for differential expression datasets. All statistically significant differentially expressed genes were included in the GO analysis to determine significant GO term enrichment (p < 0.05). All scripts and code can be found at https://github.com/ISUgenomics/2022_Kimber_Loghry_Evs_2.

The raw sequence data, from both miRNA and mRNA sequencing , have been deposited in the Genome Sequence Archive^127^ in National Genomics Data Center^128^, China National Center for Bioinformation/Beijing Institute of Genomics, Chinese Academy of Sciences (GSA: CRA010042) that are publicly accessible at https://ngdc.cncb.ac.cn/gsa.

### RT-qPCR validation of gene expression levels

1 × 10^5^ Aag2 cells were seeded in each well of a 96-well plate (Corning Inc, Corning, NY, USA) and incubated overnight at 28 °C. The following day, culture media was changed and cells were treated with either LPS (500 ng/mL) to stimulate an immune response in vitro or 1X dPBS as a negative control. Cells were incubated for an additional 12 h at 28 °C after which, culture media was changed and cells treated with tenfold serial dilutions ranging from 1 × 10^9^ to 1 × 10^2^ parasite EVs. This range was used as it allowed us to see the effects of treating cells with more EVs than would be present in a natural infection, EV levels present during a natural infection (1 × 10^6^ to 1 × 10^5^) and the effects of having fewer EVs than would occur in a natural infection. Cells were then incubated for a further 16 h at 28 °C before collection and storage in Trizol (Thermo Fisher Scientific) ahead of RNA extraction described above. cDNA was synthesized from sample RNA consisting of pooled wells of three biologically distinct samples, defined by both biological distinct cell passages and EV batches, using Superscript III First-Strand cDNA Synthesis kit (Thermo Fisher Scientific) according to manufacturer’s instructions. 20 ng of cDNA was used per qPCR reaction using Powerup SYBR green master mix (Thermo Fisher Scientific) and gene specific primers according to manufacturer’s instructions on a Quantstudio 3 Real-Time PCR system (Thermo Fisher Scientific). qPCR conditions were as follows 2 min at 50 °C, 2 min at 95 °C, 40 cycles of 15 s at 95 °C, 15 s at 59 °C, and 1 min at 72 °C. C_T_ values were averaged across technical replicates and normalized against RPS17. Primer sequences for AAEL002590 (Serine Protease) and the housekeeping gene (RPS17) can be found in Supplemental Materials 5**.**

### In vitro RNA interference

Duplexed siRNA was designed and produced targeting the serine protease gene by Integrated DNA Technologies (Coralville, IA, USA). Sequences for the duplexed siRNA can be found in Supplemental Materials 5. 4 × 10^4^ Aag2 cells were seeded per well of a 96-well plate and incubated overnight. 5 pmol of siRNA or scrambled negative control was mixed with lipofectamine RNAiMAX Reagent (Thermo fisher Scientific) to create a 1 pmol siRNA solution. 10 µL of the 1 pmol siRNA solution was added per well and incubated for 24 h. To determine RNAi efficiency, total RNA was isolated from cells for subsequent RT-qPCR as described above.

### *Aedes aegypti* injections

Four to five-day old *Ae. aegypti* (Liverpool strain) female mosquitoes were intrathoracically injected with 69 nL of LPS (1 mg/mL) [Sigma Aldrich] or 1X dPBS (Thermo Fisher Scientific) using a Nanoject III injector (Drummond Scientific Company, Broomall, PA, USA) and incubated for six h at 27 °C prior to EV injection. Mosquitoes were then challenged with serial dilutions of 1 × 10^7^, 1 × 10^6^, 1 × 10^5^ EVs, or 1X dPBS as a control. Total RNA was isolated from three biologically distinct batches of 8 mosquitoes per treatment group 24 h post-challenge. Mosquitoes were homogenized using a mortar and pestle in 1 mL of Trizol. The resulting suspension was centrifuged at 12,000 × *g* for 10 min at 4 °C to remove debris, and the supernatant collected. RNA extraction, cDNA synthesis and qPCR were performed as described above.

### Phenoloxidase activity assay

Pooled hemolymph was collected from 10 adult female mosquitoes by perfusion and prepared for PO assay as previously described^84,129,130^. Briefly, 10 µL of hemolymph was mixed with 90 µL of 3, 4-Dihydroxy-L-phenylalanine (L-DOPA, 4 mg/mL)(Sigma Aldrich) dissolved in nuclease-free water (Cytiva). After an initial 10 min incubation at room temperature, PO activity was measured at 490 nm every 5 min for 30 min, then the final activity was measured at 60 min using a Synergy HTX Multi-Mode Microplate Reader (Agilent). To determine if AAEL002590 was directly involved in the PO pathway, AAEL002590 was knocked down via siRNA in Aag2 cells as previously described. After the 24 h incubation, Aag2 cells were challenged with LPS (500 ng/mL) for either 6 or 24 h. 10 µL of either control or siRNA treated Aag2 cell culture media was mixed with 90 µL L-DOPA as described above. PO activity was allowed to proceed at room temperature overnight after which PO activity was measured at 490 nm.

### Statistical analysis

In vitro RT-qPCR validation was analyzed using a repeated measures one-way ANOVA while in vivo RT-qPCR validation was analyzed using mixed effects one-way ANOVA. Multiple comparisons were conducted using the Dunnett statistical hypothesis testing method. Enrichment of functions within the molecular function, biological process, and cellular component GO term sub-ontologies were analyzed using a Fisher’s exact test. In vivo PO assays were analyzed using a repeated measures two-way ANOVA with a Šidák multiple comparisons test while in vitro PO assays were analyzed with multiple T tests with a Holm- Šidák multiple comparison test. For all significance testing p-values < 0.05 was considered significant. All ANOVAs were completed using GraphPad prism 9.3.1 (GraphPad Software, San Diego, CA, USA).

## Supplementary Information

Supplementary Information 1.

Supplementary Information 2.

Supplementary Information 3–5.

## Data Availability

Data associated with this article can be found within the article or in the corresponding supplemental materials. Raw sequencing data has been made publicly available at https://ngdc.cncb.ac.cn/gsa with accession number CRA010042.
