# Supplementary material for: Extracellular vesicles secreted by Brugia malayi microfilariae modulate the melanization pathway in the mosquito host
Source: Sci Rep. 2023 May 31;13:8778. doi: 10.1038/s41598-023-35940-9 (PMC10232515; doi:10.1038/s41598-023-35940-9)
Supplement: Supplementary file 3 — Supplementary Information 3–5. [file 41598_2023_35940_MOESM3_ESM.pdf]

Supplemental Materials for

Extracellular vesicles secreted by *Brugia malayi* microfilariae modulate the melanization pathway in the mosquito host

Hannah J. Loghry[1*], Hyeogsun Kwon[2], Ryan C. Smith[2], Noelle A. Sondjaja[1], Sarah J. Minkler[1], Sophie Young[1], Nicolas J. Wheeler[3], Mostafa Zamanian[4], Lyric C. Bartholomay[4], Michael J. Kimber[1]

[1]Department of Biomedical Sciences, College of Veterinary Medicine, Iowa State University, Ames, Iowa, USA

[2]Department of Entomology, College of Agriculture and Life Sciences, Iowa State University, Ames, Iowa, USA

[3]Department of Biology, College of Arts and Sciences, University of Wisconsin-Eau Claire, Eau Claire, Wisconsin, USA

[4]Department of Pathobiological Sciences, School of Veterinary Medicine, University of Wisconsin-Madison, Madison, Wisconsin, USA

Supplemental Materials 1: miRNA-Seq Data Excel File

Contain full list of miRNAs identified in each treatment group, raw read counts for miRNAs mapped to both *Ae. aegypti* and *B. malayi*, and calculated fold changes and associated p-values.

Supplemental Materials 2: mRNA-Seq Data Excel File

Contain full list of differentially expressed mRNAs of LPS v LPS + *B. malayi* EV, raw read counts for identified mRNAs, and calculated fold changes and associated p-values.

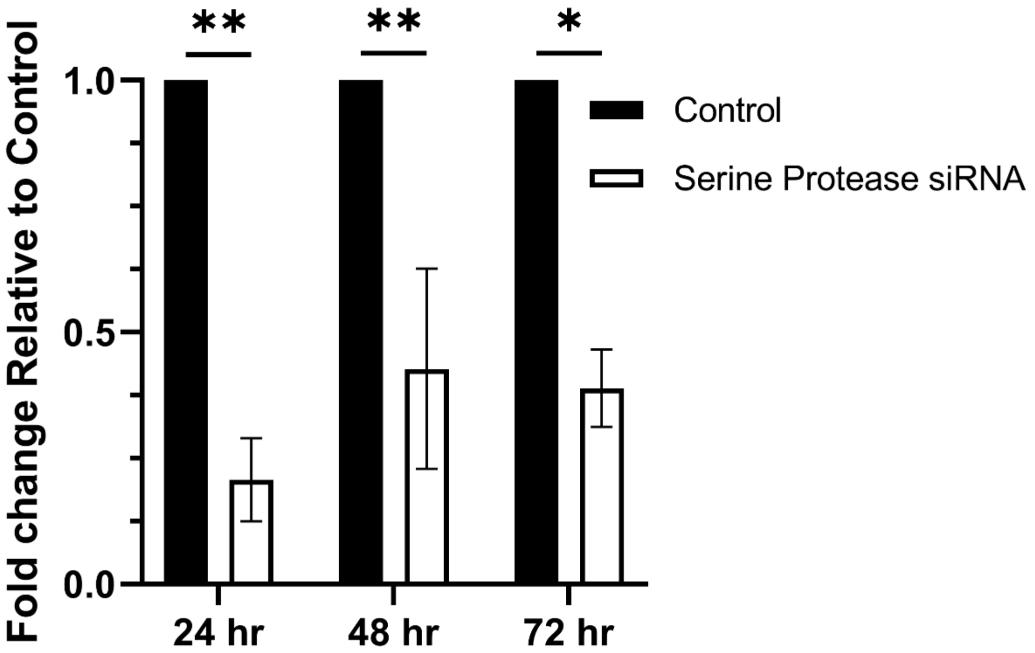

Supplemental Materials 3: Efficiency time course of RNAi knockdown of AAEL002590

Aag2 cells were treated with 1 pmol (final concentration) of duplexed siRNA. Gene expression of AAEL002590 was quantified using RT-qPCR. Expression of AAEL002590 was significantly reduced 79%, 57%, and 61% at 24 h, 48 h and 72 h post-treatment, respectively, as compared to control cells. Knockdown was the most efficient 24 h post-treatment therefore this time point was used in all experiments going forward. N = 3 (minimum). Mean ± SEM. *P < 0.05, **P < 0.01.

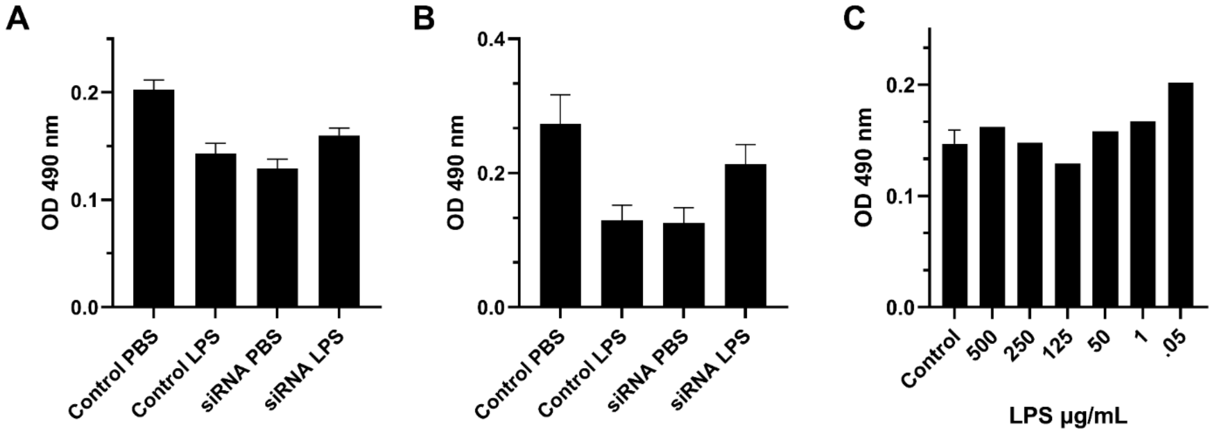

Supplemental Materials 4: LPS did not strongly induce PO activity in Aag2 cells

Aag2 cells were treated with either 1X dPBS or LPS (500 ng/mL) to test the effects of knockdown of the serine protease gene on PO activity *in vitro*. Both six h post-treatement (A) and 24 h post-treatment (B) LPS failed to induce PO activity as expected. A dose response experiment of various LPS concentrations (C) showed that no concentration of LPS was strongly inducing PO activity.

Experiment	Gene	Forward Primer	Reverse Primer
RT-qPCR gene expression validation			
	AAEL002590	AGAGCCTAGTTGCGTTGTTAG	CTGTACTGACTTCTGTGGGAAC
	Housekeeping Gene (RPS17)	CACTCCCAGGTCCGTGGTAT	GCACACTTCCGGCACGTAGT
RNAi			
	Duplexed siRNA for AAEL002590	AAAGAUAUCAUUGCUAGUGACCAAA	UCUUUCUAUAGUAACGAUCACUGGUUU

Supplemental Materials 5: Primer Sequences

Primer sequences for RT-qPCR validation of AAEL002590 (serine protease). RT-qPCR data were normalized against the housekeeping gene RPS17. Duplexed siRNA for knockdown of AAEL002590 was produced by Integrated DNA Technologies with the provided.
